# Paraprobiotics: A New Perspective for Functional Foods and Nutraceuticals

**DOI:** 10.3390/nu13041225

**Published:** 2021-04-08

**Authors:** Rosa Anna Siciliano, Anna Reale, Maria Fiorella Mazzeo, Stefano Morandi, Tiziana Silvetti, Milena Brasca

**Affiliations:** 1 Institute of Food Sciences, National Research Council (CNR-ISA), Via Roma 64, 83100 Avellino, Italy; rsiciliano@isa.cnr.it (R.A.S.); anna.reale@isa.cnr.it (A.R.); 2 Institute of Sciences of Food Production, National Research Council (CNR-ISPA), Via G. Celoria 2, 20133 Milan, Italy; stefano.morandi@ispa.cnr.it (S.M.); milena.brasca@ispa.cnr.it (M.B.)

**Keywords:** paraprobiotics, probiotics, dairy foods, non-dairy food, health benefits, inactivation, immunomodulation

## Abstract

Probiotics are live microorganisms that confer health benefits on the host. However, in recent years, several concerns on their use have been raised. In particular, industrial processing and storage of probiotic products are still technological challenges as these could severely impair cell viability. On the other hand, safety of live microorganisms should be taken into account, especially when administered to vulnerable people, such as the elderly and immunodeficient individuals. These drawbacks have enhanced the interest toward new products based on non-viable probiotics such as paraprobiotics and postbiotics. In particular, paraprobiotics, defined as “inactivated microbial cells (non-viable) that confer a health benefit to the consumer,” hold the ability to regulate the adaptive and innate immune systems, exhibit anti-inflammatory, antiproliferative and antioxidant properties and exert antagonistic effect against pathogens. Moreover, paraprobiotics can exhibit enhanced safety, assure technological and practical benefits and can also be used in products suitable for people with weak immunity and the elderly. These features offer an important opportunity to prompt the market with novel functional foods or nutraceuticals that are safer and more stable. This review provides an overview of central issues on paraprobiotics and highlights the urgent need for further studies aimed at assessing safety and efficacy of these products and their mechanisms of action in order to support decisions of regulatory authorities. Finally, a definition is proposed that unambiguously distinguishes paraprobiotics from postbiotics.

## 1. Introduction 

Since the first observation by Metchnikoff more than 100 years ago, the popularity of probiotics boosted substantially. In the last decades, particularly in the last five years, a large body of experimental and clinical evidence on the health benefits of probiotics has appeared [1]. Their biological effects include disease treatment (i.e., restoration of health), disease prevention (i.e., preservation of health) and health “optimization” [2]. The ongoing interest in probiotic bacteria goes hand in hand with a rapid and lucrative expansion of the sector of functional foods and supplements containing these bacteria. However, a rigorous evaluation and validation of health and/or functionality claims along with safety and practical use aspects remains a critical issue for the field of probiotic and functional food [3]. 

According to the earlier revised definition by the International Scientific Association for Probiotics and Prebiotics (ISAPP), probiotics are “live microorganisms that, when administered in adequate amounts, confer a health benefit on the host” [4]. Thus, probiotic cultures should be formulated in such a way that they can reach the target site in the host after surviving throughout processing, storage and gastrointestinal transit while remaining highly viable and in sufficient numbers. Nevertheless, there is still no consensus what an adequate intake of live microorganisms is [1,5]. Moreover, concerns about probiotic adverse effects, especially for at-risk groups, such as immunocompromised individuals, people with an abnormal gastrointestinal mucosal barrier, patients following surgical treatments or premature newborns, have been raised. If present in high concentration, probiotics can negatively influence the balance between anti- and proinflammatory cytokines as well as other cellular functions, causing altered long-term immune responses in subjects with immune disorders [6,7]. The European Food Safety Authority (EFSA) has registered cases of *Lactobacillus rhamnosus* sepsis associated with probiotic therapy. A study regarding 89 patients with *Lactobacillus* bacteremia reported the mortality rate of 26% within 1 month and 48% within 1 year following infection onset. Even if rare, endocarditis due to *Lactobacillus* infection results in a high mortality rate, averaging 30%. A randomized controlled trial Probiotic prophylaxis in patients with predicted severe acute pancreatitis (PROPATRIA) highlighted a significantly increased mortality (16% vs. 6%) due to bowel ischemia (9 vs. 0) among severe acute pancreatitis patients subjected to probiotic administration [8]. A systematic review of articles published between 1976 and 2018 pointed out 93 cases of patients who developed infections as a consequence of probiotic ingestion. *Saccharomyces* was the most frequent genus with 47 cases, followed by *Lactobacillus* (26 cases), *Bifidobacterium* (12 cases), *Bacillus* (5 cases), *Pediococcus* (2 cases) and *Escherichia* (1 cases), respectively [9]. Bacteremia and fungemia represent the most frequently reported ailments, but the list is set to expand in the near future. The possible horizontal transfer of genes from pathogenic bacteria in the gut is another critical issue due to the risk of development and spread of virulence traits and antibiotic resistance [1,10,11,12]. Other doubts could be raised about the probiotic mechanism of action, their strain-specific properties and their being in competition with commensal gut microflora for colonization [13]. Viability and safety are relevant challenges for the probiotic industry. The scientific community and regulators ought to clear up doubts surrounding probiotic preparations, especially considering that the next generation of probiotics comprising new species being used for this intended purpose without a long history of use (i.e. *Akkermansia muciniphila*, *Faecalibacterium* and *Bacteroides* species) will keep being launched more and more often [1,14].

All these drawbacks related to the administration of viable microorganisms led to the interest in non-viable probiotic preparations. Since 2004, increasing evidence has been suggesting that some health benefits of physiologically active bacteria are not strictly associated with their viability. In fact, probiotic products also contain dead cells, which can produce a biological response as effectively as their live equivalents, highlighting the fact that probiotic products may be further used beyond their expiry. This is called the “probiotic paradox” (or, as some authors have suggested, the “probiotic advantage”), i.e., both live and dead cells can produce a biological response. Though there may be a potential benefit from the consumption of dead microorganisms, they cannot be classified as probiotic [15]. Hence, the term “paraprobiotic” together with a wide range of synonyms has been coined. According to the most recurrent definition, paraprobiotics, also known as non-viable probiotics, inactivated probiotics, tyndallized probiotics or ghost probiotics, are “non-viable microbial cells (either intact or broken), or crude cell extracts, which, when administered (orally or topically) in adequate amounts, confer a benefit on the human or animal consumer” [13,16].

Although the molecular mechanisms underlying paraprobiotic action still need a thorough investigation, scientific evidence has shown that, similarly to probiotics, molecules present on the cell surface (peptidoglycan, teichoic acid, cell wall polysaccharides, cell surface-associated proteins, etc.) could constitute the first line of interaction between paraprobiotics and the host, thus mediating the beneficial effects [17].

Paraprobiotics have been proven to modulate anti-inflammatory and positive immune responses in animals and humans, with some advantages if compared to probiotics. Non-viable microbial cells may exhibit enhanced safety, i.e., reduced risk of sepsis and antibiotic resistance, as well as technological and practical benefits, i.e., longer shelf life, since the cold chain is not required for microorganism viability and stability. These features also enable their application in underdeveloped regions [7,18].

Another great advantage is no loss of bioactivity when administered in combination with antibiotics or antifungal agents [19]. Killed probiotics also offer an attractive solution to overcome problems correlated to formulation of the food matrix [20]. Furthermore, research on gut microbiota brought about newly recognized bacteria from the gastrointestinal tract providing beneficial effects for human physiology, as mentioned above, and paraprobiotic preparations could be useful for solving complications related to stability during commercialization and safety of these next-generation probiotics since they are often strictly anaerobic bacteria; thus, their production and stability represent major challenges. Interestingly, besides the most studied probiotic genera *Lactobacillus* and *Bifidobacteria* that have been awarded the GRAS (generally recognized as safe) and QPS (qualified presumption of safety) status for intentional addition to food and feed by the FDA and EFSA respectively [11], other probiotic agents (e.g. *Escherichia coli*, *Bacillus*, *Saccharomyces*) and next-generation probiotics that need to be studied for their safety profile (*Faecalibacterium prausnitzii* and other members of Ruminococcaceae, *Bacteroides*, *Clostridium* XIVa cluster bacteria, and *Akkermansia* spp.) are emerging [13,20]. There is also evidence about anti-inflammatory and anti-allergic activity exerted by acetic acid bacteria in foods (e.g., nata de coco, kombucha and fermented milk), but it is not clear whether the live or dead cells are responsible for these beneficial effects [21].

**Figure 1:**
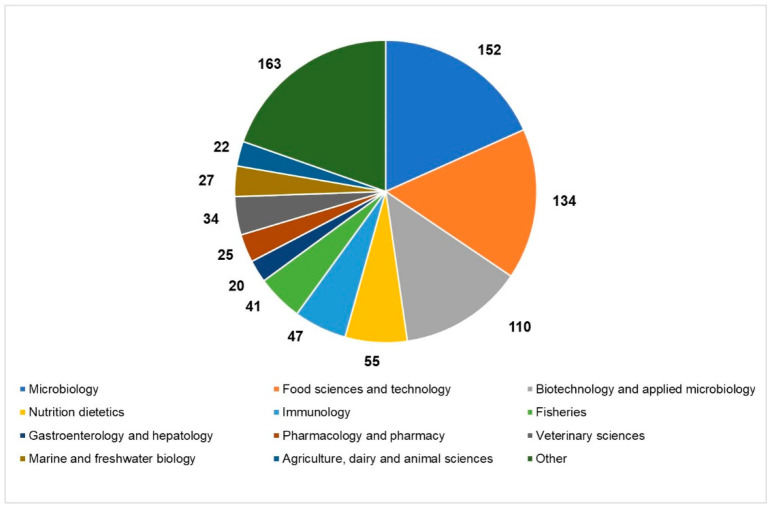
Current state of the scientific literature on paraprobiotics: distribution of papers published in the last 20 years in the main research areas (source: Web of Science; 2011–2020; updated to 22 December 2020).

An overview of the current state of the scientific literature setting “paraprobiotics” or “inactivated probiotics” as search terms, which revealed a hike in the number of articles published in the last ten years in different research areas, helps us to realize the growing interest in the inactivated microbial cells (Figure 1).

## 2. Technological Features in the Production of Paraprobiotics

Industrial processing and storage of probiotic products still represent technological challenges as these could severely impair probiotic cell viability, putatively a key requisite for the probiotic effects. As a matter of fact, probiotic products actually contain viable and non-viable cells that could both contribute to the beneficial effects on human health [22,23,24]. On the other hand, several concerns have been raised for the functionality and safety of live microorganisms in foods, especially when administered to vulnerable people, such as the elderly and immunodeficient individuals [25].

The use of paraprobiotics allows overcoming several of these drawbacks and opens up new perspectives in the design of novel functional foods, significantly simplifying industrial handling and marketing. In fact, paraprobiotics could be added to several foods that, due to their chemical or physical properties, do not offer a suitable environment for the survival of probiotics (such as fruit juice). Furthermore, paraprobiotics do not directly interact with food matrices and do not modify their organoleptic features [26]. In addition, they could be added before thermal processing of food without completely impairing health-promoting features while assuring food safety and are not affected by antibiotic treatments [27]. 

On the other hand, it should be taken into consideration that inactivated cells should be unable to produce secreted metabolites (such as bacteriocins, lactic acid, vitamins, etc.) and enzymes which could have a relevant role in the probiotic health effects. 

Due to these features, novel foods containing paraprobiotics are less affected by storage and transport conditions, thus guaranteeing a prolonged shelf life and assuring economic advantages. These points could offer an important opportunity to prompt the functional food market with safer and more stable products. 

Inactivation of probiotic cells can be achieved using physical or chemical treatments capable of modifying microbial cell components (cell membranes and envelopes, proteins, DNA, etc.) and physiological functions (enzyme activities and membrane selectivity) without completely destroying the cell structure. 

Conventional and emerging technologies for the production of paraprobiotics comprehensively reviewed by de Almada et al. [27] include those already applied for bacterial inactivation for safety purposes such as thermal processes [28,29], irradiation [30], UV rays [31], high pressure [32] and ultrasound [33]. Furthermore, a combination of techniques could result in more effective inactivation protocols [34]. These processes could specifically target different cell components and/or functions or generally damage the entire cell structure.

In particular, thermal treatments that are still the most widely used processes for producing paraprobiotics at laboratory and industrial levels [35] damage the cell membrane thus provoking leakage of nutrients and ions and cause ribosome aggregation, protein denaturation and DNA breakage [27]. As an alternative to conventional heat processes, ohmic heating has been very recently proposed for paraprobiotic production. This emerging technology involves the passage of alternating electric current through the sample, thus leading to a fast and uniform heating. Bacterial inactivation is thus caused both by thermal and non-thermal damage, such as electroporation caused by the electric field that increases the membrane permeability, inducing cell death. This technology has been applied and optimized for the inactivation of probiotic *Lactobacillus acidophilus*, *Lacticaseibacillus casei*, and *Bifidobacterium animalis* [36].

Similarly, high hydrostatic pressure and high-pressure homogenization treatments also used in combination with thermal processes can cause membrane rupture due to shear stress, as well as alteration of ribosomes and irreversible protein denaturation and coagulation leading to the inactivation of biological functions mediated by enzymes, extensive loss of solute and reduction of intracellular pH [37,38].

More recently, the application of high-intensity ultrasound (HIUS) in the inactivation of probiotics has been reviewed [39]. The effect of this technology on microorganisms is associated with physical forces generated by acoustic cavitation that cause cell wall shearing, free radicals, DNA damage and, eventually, membrane breakdown and cell lysis [34,39,40].

**Figure 2:**
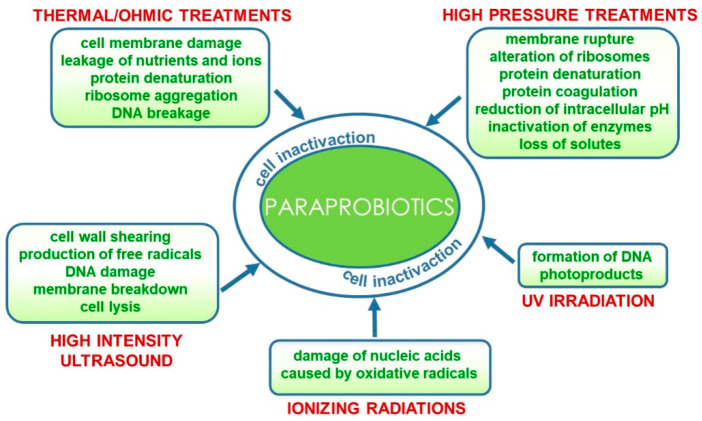
Technological processes for the production of paraprobiotics and their effects on bacterial cells.

On the contrary, other inactivation methods more specifically target particular cell components. Microbial inactivation by ionizing radiation (gamma rays or X-rays) is mainly due to the damage of nucleic acids caused by oxidative radicals originating from the radiolysis of water [41]. Similarly, UV irradiation of microbial cells induces formation of DNA photoproducts such as the pyrimidine dimer, thus interrupting both DNA transcription and translation [42]. These inactivation methods are currently suitable for producing paraprobiotics at the laboratory scale; however, further studies are needed to develop technological processes for the industrial scale up of paraprobiotics production that would preserve the beneficial effects while being time- and cost-effective [43] (Figure 2).

A sound body of scientific evidence shows the beneficial effects of paraprobiotics on human and animal health, thus definitively assessing that cell vitality is not an absolute prerequisite for the health effects [44]. However, methods and process parameters for paraprobiotic production should be carefully tailored taking into consideration the characteristics of both target microbial species and food matrices to assure that products retain their efficacy. In fact, inactivation methods could affect the beneficial effects, and paraprobiotics obtained with different technologies could exhibit different functional features. 

The ability to modulate the adaptive and innate immune systems represents the key feature of the paraprobiotic action [16,22]. Intriguingly, probiotic and paraprobiotic cells of the same species can induce similar immunological responses by triggering the same pathways or different mechanisms of action. For instance, UV-inactivated and live *L. rhamnosus* GG cells were equally effective in decreasing IL-8 production in the intestinal epithelium cells (Caco-2 cells), but their mechanisms of action involved different pathways [31]. 

Aggregation and adhesion are also important properties of probiotics, being involved in gut colonization and antagonistic effect against pathogens. These features could be affected by experimental conditions used for cell inactivation. Ostad et al. demonstrated that both live and heat-inactivated forms of fecal *L. acidophilus* (treated at 60 °C for 30 min) were able to inhibit the attachment of pathogenic bacteria (*Escherichia coli* and *Salmonella typhi*) to Caco-2 cells [45]. Similarly, Tareb et al. showed that heat-killed *L. rhamnosus* CNCM I-3698 and *Lactobacillus farciminis* CNCM I-3699 (autoclaved at 120 °C for 15 min) exhibited co-aggregative abilities toward *Campylobacter jejuni* and higher exclusion potential against binding of this pathogen to mucin compared to the live counterparts. The ability of inactivated cells to adhere to different intestinal matrix models (as evaluated by qPCR) was also reported [46].

More recently, Singh et al. investigated the adhesion and antagonistic activity of several probiotic *Lactobacillus reuteri* strains (live, heat-inactivated and treated with 5 M LiCl) toward select pathogens. The reported data highlighted that these properties were strictly strain-specific. The inactivated cells (80 °C for 10 min) adhered to Caco-2 cells, although to a slightly lesser extent compared to the live counterparts, while the pathogen inhibition abilities were significantly reduced. The adhesion and antagonistic potential of the probiotic strains were lost upon exposure to 5 M LiCl, thus indicating the involvement of surface proteins [47].

As a matter of fact, morphological changes on several heat-treated lactic acid bacteria strains were observed by field-emission scanning electron microscope (FE-SEM). In particular, resulting cell surfaces of all the heat-treated bacteria were rougher and more uneven than those of viable untreated cells. This finding was accompanied by a decrease of the adhesive ability of heat-killed bacteria with increasing temperature. Interestingly, heat treatment decreased the adhesion ability but did not affect the immunostimulatory activity [28].

However, further studies are required to definitively assess whether paraprobiotics retain their adhesion and pathogen exclusion abilities and clarify how these features are affected by the inactivation processes, applying the most up-to-date methodologies. It should also be kept in mind that models used to assess cell adhesion in vitro only represent simplifications of in vivo conditions and the counting of truly adherent paraprobiotic cells could still present several experimental difficulties [48].

Another interesting feature of probiotics is their ability to remove cholesterol from media via several possible mechanisms including assimilation during growth and incorporation into the cell membrane [49,50]. It has been reported that sonication-killed cells of *Bifidobacterium longum* SPM1207 isolated from healthy adults and orally administrated to rats retained the ability of lowering cholesterol, blocking the body weight increase and relieving or eliminating constipation in rats, as also shown for the viable probiotic cells [33]. However, Lye et al. showed that, although low-intensity ultrasound treatment increased viability and cholesterol removal ability of lactobacilli, a decrease in both these features was observed for higher-intensity ultrasound treatment (100 W for 3 min), thus suggesting that the ability of lactobacilli cells to assimilate cholesterol could be partly associated with the growth ability [51].

Detailed studies aimed at investigating the molecular mechanisms at the basis of the exhibited health effects of paraprobiotics and performing comparisons with viable counterparts are still required and crucial to set up inactivation protocols that preserve their beneficial action. 

## 3. Analytical Techniques for the Quality Control of Paraprobiotic-Containing Products and Regulatory Aspects

### 3.1. Paraprobiotics Detection and Quantification

Paraprobiotics are defined as “inactivated microbial cells (non-viable) that confer a health benefit to the consumer” [27]. Considering this definition, it is easy to understand that is not possible to enumerate the paraprobiotics using classical microbiology methods based on the ability of the single cells to grow and form colonies. The cultivation technique also does not provide information about cell integrity and metabolic activity, but damaged and injured cells can still retain some metabolic activity that contributes to health promotion [27]. On the other hand, culture-independent methodologies, such as polymerase chain reaction (PCR) techniques, are able to detect viable cells or the overall microbiota of different matrices (living and dead cells), but fail in the selective quantification of non-viable cells. Moreover, the inactivation methods to produce paraprobiotics could cause DNA damage that would negatively affect PCR results.

Flow cytometry is a potential analytical technique that holds the potential to quantify non-viable cells in a matrix. Over the last 20 years, flow cytometry has gained increased popularity in microbiological research since it allows the determination of viable bacteria but also the enumeration of damaged/dead cells [52]. This technique was initially developed for studying eukaryotic cells but is currently used to detect and explore the physiological state of prokaryotic cells in foods and probiotic products [53,54].

The flow cytometry principle is based on dual nucleic acid staining with a cell-permeant dye (thiazole orange, SYTO 9 or SYTO 24) and a cell-impermeant dye (propidium iodide). Thiazole orange or equivalents permeates membranes of total cells and stains the nucleic acids with green fluorescence. Propidium iodide penetrates only bacteria with damaged membranes, causing a reduction in thiazole orange fluorescence when both dyes are present. Thus, live cells with intact cell membranes fluoresce bright green, bacteria with slightly damaged membranes exhibit both green and red fluorescence, whereas bacteria with broken membranes fluoresce red [52,53]. The main advantages of this technique are as follows: short assay and data generation times (1–2 min), minimum sample volume (from 5 μL), detection of live and dead cells and less labor compared with conventional plating techniques (Wilkinson, 2020). Recently, flow cytometry was applied to characterize a multi-strain probiotic product. This technique allowed the authors to quantify non-viable cells in the overall population of analyzed samples, highlighting that flow cytometry could be a powerful tool to enumerate paraprobiotics cells in food matrices [53]. 

However, it has to be considered that by using flow cytometry, it is possible to assess the content of cells and their viability, but no taxonomical information on the microorganisms that are present can be obtained. Since the concept of “probiotic” also requires the identification of probiotic microorganisms in the final product, flow cytometry should be used as a complementary method along with PCR methods to allow paraprobiotics detection, count and identification. In addition, a study of Klein et al. showed the suitability of a proteomic approach based on capillary electrophoresis coupled to mass spectrometry (CE/MS) for quality control of inactivated probiotics preparations [55].

Soejima et al. have recently launched digital PCR as a robust tool for the routine analysis of heat-killed lactobacilli-supplemented foods [56]. This assay targets multiple copies of the 16S rDNA without being affected by several DNA recovery rates in the same sample. Hence, it seems to be useful to guarantee accurate cell supplementation of nutritional foods, thereby also avoiding a cell content higher by tenfold and, consequently, excessively high production costs.

### 3.2. Regulatory Aspects

Despite favorable perspective on the use of paraprobiotics, several aspects of this concept are not fully understood yet. The term “paraprobiotic” is misleading by itself, for it suggests, if literally taken, that it is effective only if administered in the presence (“para”, i.e., “side by side”) of a probiotic. The proliferation of overlapping terminology and the absence of a universally recognized definition induce vagueness, which makes challenging communication both between researchers and of the concept to the consumer. The current situation requires a consensus panel in order to draw attention to the confusion that reigns within the probiotic glossary and to address the emerging terms in the “biotics” field with the objective of establishing a generally agreed terminology. Some confusion also derives from the definition of paraprobiotics that includes non-viable intact or broken cells (i.e., cell lysates or fragments, cell membrane or cell wall components) or the cellular extract. This entails a partial overlap with the term “postbiotics”, defined as extracts of non-viable probiotics and comprising cell membrane components such as surface proteins, lipopolysaccharides, teichoic acids, etc. [16,57].

**Figure 3:**
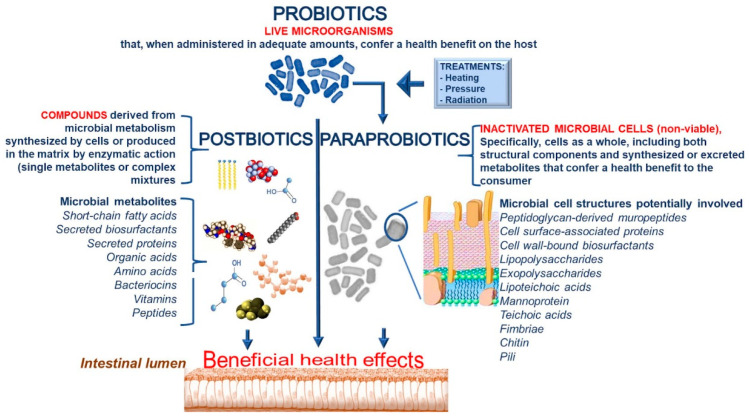
Definitions of probiotics, paraprobiotics and postbiotics together with cell components involved in biological activities.

We propose defining the term “paraprobiotic” as “inactivated microbial cells (non-viable), specifically, cells as a whole, including both structural components and synthesized or excreted metabolites that confer a health benefit to the consumer.” In addition, we recommend defining the term “postbiotics” as “compounds derived from microbial metabolism synthesized by cells or produced in the matrix by enzymatic action.” Postbiotics can be single metabolites or even very complex mixtures. A detailed definition of the different terms and the cell components involved in the biological activities are reported in Figure 3.

There are several key points to be clarified in order to support regulatory authorities for defining the requirements for the registration and approval of foods and dietary supplements containing paraprobiotics. In addition to a punctual and unequivocal definition recognized at the international level, specific attention is required with regard to paraprobiotics production methods, quality control criteria, how to detect and quantify their presence and how to assess their safety and efficacy.

Means of inactivation may affect the physiological activity of the resulting dead cells and the stability of their beneficial effects during shelf life anyway. This is another aspect to deepen and clarify to make the best possible use of paraprobiotic opportunities [27].

## 4. Paraprobiotics for the Production of Functional Dairy Products

Given that the viability of probiotics in food is a critical factor, some food matrices are more suitable to deliver probiotics. The food carrier also affects bacterial susceptibility to harsh gastrointestinal conditions (acidity, bile and various enzymes), ability to adhere to intestinal epithelial cells and immunomodulatory properties. Since the buffering capacity of milk and milk fat assures probiotic survival through processing and storage, dairy products, including yoghurt and other fermented milks, cheese and frozen fermented dairy desserts, represent an ideal and marketable carrier of probiotic bacteria to consumers [58,59].

Particularly, yoghurt is a very efficient probiotic vehicle compared with other food products, but also ice cream, which is rich in milk fat, is effective in enhancing microbial survivability and acid tolerance [60,61]. Nevertheless, the extension of shelf life is limited in probiotic yoghurt due to the oxidative stress suffered by probiotic bacteria [27]. Many cultured dairy products, then, fail to meet the first criterion for probiotics, i.e., “containing live microorganisms” at consumption time, because probiotic strains cannot endure the acidity of the product, which may also increase over time when it contains lactic acid-producing bacteria. In addition, foods of animal origin may represent a reservoir of antibiotic-resistant genes transmissible to the gut microbiota. Commercial antibiotic-resistant probiotic strains, mostly *Lactobacillus* and *Bifidobacterium*, have been actually found in milk culture, yoghurts and cheese [62].

Paraprobiotics could be of particular interest for the dairy sector, since they remain stable in a wide range of pH and temperature, allowing the incorporation into foods with high acidity and before thermal processing without loss of functionality. Their addition does not change the sensory properties of the product, thus avoiding detrimental modifications like high acidification after fermentation in yoghurt [35]. Actually, yoghurt naturally contains paraprobiotics, but they cannot be controlled, and this does not ensure an effective physiological response in vivo [43].

Up to now, only a few works have addressed the potential application in the dairy industry of paraprobiotics added or generated during processing, and clinical studies have been performed to investigate the efficacy of the products containing paraprobiotics and their health effect at the physiological level (Table 1).

Some studies reported that paraprobiotics affect behavior under chronic or prolonged stress situations. The daily consumption of paraprobiotic *Lactobacillus gasseri* CP2305 (190 g fermented milk beverage, 1 × 10^10^ heat-treated bacteria, for five weeks) has been proven to improve sleep quality and normalize bowel habits in subjects under stressful conditions [63]. The same paraprobiotic strain (administered once daily for three weeks) showed a beneficial effect on the regulation of intestinal function in constipated persons [64] and it was also able to alleviate clinical symptoms in patients with irritable bowel syndrome [65].

A yoghurt containing paraprobiotic cells derived from *Lactobacillus delbrueckii* subsp. *bulgaricus*, *Streptococcus thermophilus*, and *Lb. acidophilus* has also been recognized to prevent impaired barrier function in gut epithelium in human intestinal Caco-2 cells [66]. 

Non-viable bacteria in fermented milk are also useful to modulate gut microbiota. A clinical study conducted on young children fed with a cow’s milk fermented product containing the heat-killed probiotic strain *Lactobacillus paracasei* CBA L74 showed an increase in butyrate producers (*Oscillospira* and *Faecalibacterium*), *Bacteroides* and different *Roseburia* and *Blautia* oligotypes [67].

A clinical study by Liu et al. provided evidence that pasteurized yoghurt with no viable lactic acid bacteria consumed twice daily for seven weeks was effective in improving constipation symptoms and intestinal health [68].

Nongrowing and dead cells of *Lactococcus lactis* inactivated upon pasteurization applied to fermented milk have been reported to possess antihypertensive potential and to remove cholesterol, probably via cholesterol attachment to their cell surface thanks to the chemical and structural properties of the cell wall peptidoglycans [69].

Finally, although the high-intensity ultrasound (HIUS) technology showed the potential to generate paraprobiotics while improving the fermented dairy product processing (e.g., enzyme release and carbohydrate hydrolysis), there are no studies focusing on the contribution of paraprobiotics in sonicated dairy products [39].

## 5. Paraprobiotics vs. Probiotics in Functional Non-dairy Products and Nutraceuticals

Although dairy products are currently the most common food carrier to deliver probiotics and paraprobiotics, an increasing number of non-dairy food matrices is attempted as potential for delivery of these microbial components. In fact, in recent years, the increasing health concerns regarding dairy products resulted in a shift towards non-dairy foods such as cereal-based products, fruit and vegetable drinks and ice cream [70,71].

There are several reasons why people might be looking for substitutes of probiotic and paraprobiotic dairy products, such as milk protein allergy, as 2–3% of children under three have a milk allergy [72]; lactose intolerance, the most common type of carbohydrate malabsorption that is associated with the inability to digest lactose into its constituents due to low levels of the lactase enzyme [73]; high cholesterol content and high amounts of saturated fatty acids in dairy-based foods [74]; increased vegan or vegetarian diets attributed to health consciousness in the general population [75]; major attention to the presence of potential contaminants in conventional milk and dairy products, including hormones, pesticides and antibiotics [76]. 

These concepts are even more important for the elderly people that may have multimorbidity and diverse risk factors. Considering that life expectancy has increased rapidly in the recent years as well as the cost of medical care, the development of new non-dairy functional foods becomes essential to promote healthy diets and improve life quality. Therefore, both probiotics and paraprobiotics could be extensively used in functional non-dairy foods, medicine, supplements and fodder [10,77].

Nowadays, functional foods containing probiotics represent an industrial sector with growing market shares and large commercial interest [78]. However, preparation of a probiotic product presents many challenges related to microbial growth, survival, viability, stability and functionality in food processing, storage and consumption. In particular, the application of probiotic cultures in non-dairy products represents a great challenge since probiotic cultures included as ingredients in these products, do not usually multiply, which sets great demands on the probiotic stability. Factors like water activity, oxygen tension and temperature become increasingly important when dealing with these kinds of products. Storage at room temperature, which is common for many types of non-dairy products, such as cereal products, beverages, confectionary and so on, can create an overwhelming challenge for probiotic stability [79]. Consequently, adding probiotics to fruit- and cereal-based matrices is more complex than formulating dairy products.

To overcome these disadvantages, paraprobiotics can be added as ingredients in non-dairy products. Their application in foods and beverages could comprise an important alternative for specific cases in which probiotics are damaged and do not survive during processing and/or shelf life, although these aspects are poorly explored and deserve thorough investigation.

At the clinical level, there is currently an increasing interest in the use of heat-killed preparations of different probiotic strains in the management of intestinal, allergic respiratory and topical diseases and also as support in *Helicobacter* therapy [80]. So far, the main exploitations have been done on paraprobiotics as supplements (Table 1). More than fifty paraprobiotic capsules or tablets obtained by heat/UV/sonication treatment of lactic acid bacteria, *Saccharomyces* or *Bifidobacterium* probiotic strains, that provide health benefits when administered with diet were recorded by de Almada et al. [27].

Murata et al. confirmed the safety of continuous oral intake of a powder containing about 1×10^10^ heat-killed *L. paracasei* MCC1849 in healthy young adults [81]. In addition, the effects of inactivated cells of *Bifidobacterium longum* on obesity and blood glucose levels in an obese diabetes murine model were evaluated by administering inactivated *Bifidobacterium* cells orally for five weeks. The treated mice showed a significant decrease of body weight gain, adipose tissue mass and blood glucose levels, as well as reduced levels of cholesterol and triglycerides [82].

Currently, some paraprobiotic products with proven effects in the prevention or treatment of some diseases are commercially available as supplements [43]. Many of them contain non-viable or lyophilized heat-treated lactic acid bacteria that were proven to have healthy effects such as to be effective in controlling *Helicobacter pylori* infection [83,84] or in the treatment of diarrhea [85,86].

To date, applications of paraprobiotics added or generated during the processing of non-dairy foods are very scarce (Table 1). One of the few examples of paraprobiotics used in non-dairy products is the *L. gasseri* CP2305 strain inactivated by heat. The daily consumption for 12 weeks of sports drinks containing heat-inactivated *L. gasseri* CP2305 contributed to recovery from fatigue and alleviation of anxiety and depressive moods in young athletes facing stressful situations [87]. Furthermore, this paraprobiotic is also effective when administered to humans in different matrices. In fact, daily consumption for three weeks of a corn syrup-based beverage containing the same paraprobiotic (heat-inactivated) by healthy subjects with low or frequent bowel movements beneficially affected intestinal functionality [88]. The consumption of the same beverage led to improvements in negative stress-related behaviors in a group of medical students subjected to stressful situations as evaluated by psychological responses and biological parameters associated with stress [89]. Very recently, Barros et al. proposed a whey-grape juice drink containing paraprobiotic *Lacticaseibacillus casei* 01 inactivated by ohmic heating. Interestingly, this drink exhibited hypoglycemic activity in vitro (through the inhibition of α-glucosidase and α-amylase) and was effective in reducing postprandial glycemia in healthy individuals [90].

Moreover, for the widespread application of paraprobiotics in foods, a few conditions should be addressed, such as ease of production, good solubility in foods, lack of or reduced interaction with foods and stability during processing and storage. Last but not least, the elucidation of the mechanisms of action of paraprobiotics, including the determination of specific active compounds responsible for the beneficial effects, comprise key information to enable not only the application of paraprobiotics in foods, but also their regulation by health authorities [27]. 

## 6. Beneficial Health Effects of Paraprobiotics

Modulation of the host’s adaptive and innate immune system is a key mechanism underlying the beneficial effects exerted by probiotics on animal and human health, in particular in the prevention and treatment of pathologies such as atopic dermatitis, gastrointestinal and respiratory diseases, virally induced infectious diseases, infections and allergies.

Innate immune cells, including neutrophils, monocytes, macrophages and natural killer cells, represent the first defense system activated by the host to face off with infection by pathogens, even if these cells are only slightly specific in the recognition of their targets. However, they provide an interface between innate and adaptive immune response as they act as professional antigen-presenting cells (APCs), thus promoting the specific immune response that involves different subsets of T helper cells (Th1, Th2, and Th17 cells), regulatory T cells and B cells. As innate and adaptive immunity are deeply interconnected, probiotics have a multifaceted role in immunomodulation. The reported effects exerted by probiotics on host immune response include, among others, modulation of the profile of secreted cytokines, both proinflammatory (interleukin 12 (IL-12), IL-3, tumor necrosis factor alpha (TFN-α), interferon gamma (IFN-γ), etc.) and anti-inflammatory (IL-10, IL-6, IL-13, IL-4); stimulation of the production of immunoglobulins (IgE, IgA IgM, IgG1) by B cells; inhibition of the signaling pathways such as the nuclear factor-κB pathway [16,94,95,96].

The innate immune response depends on transmembrane pattern recognition receptors (PPRs) such as Toll-like receptors (TLRs) of host epithelial and immune cells (neutrophils, monocytes, macrophages, dendritic cells, natural killer cells) that link microbial-associated molecular patterns (MAMPs) present on the microorganisms and intracellular nucleotide-binding oligomerization domain (NOD) proteins that sense MAMPs in the cytoplasm and recognize pathogens that replicate intracellularly [97,98]. As a matter of fact, these mechanisms do not require cell viability as they could be also induced by extracellular bacterial molecules, cell wall constituents (peptidoglycans, teichoic acids, proteins) and structural features of the cell envelope including the S-layer, capsule of polysaccharides and pellicle [99,100]. These findings open the way to the use of paraprobiotics as health-promoting agents, taking into account their safety benefits especially for immunocompromised subjects [25,101,102,103]. 

Several studies investigated the impact of paraprobiotics on the immune system response in comparison with their probiotic counterpart mainly in different cell lines. For instance, Chuang et al. examined the effects of three heat-killed *Lactobacillus* strains on mouse splenocyte proliferation and the activation of mouse dendritic cells, demonstrating their efficacy in modulating interleukin secretion [104]. Lopez et al. demonstrated that viable and UV-inactivated *Lactobacillus rhamnosus* GG cells both reduced the production of IL-8 upon flagellin induction in human epithelial colorectal adenocarcinoma Caco-2 cell line [31]. It has been reported that live and heat-killed cells of *L. rhamnosus* ATCC 7469 were both able to induce the synthesis of different cytokines with proinflammatory (TNF-α and IL-6) or regulatory (IL-10) functions in mouse macrophages [105]. More recently, the antioxidative activity and immune-stimulating potential of heat-killed *L. brevis* KCCM 12203P cells in comparison with their viable counterpart have been assessed [106] and the effects of live and heat-inactivated *B. animalis* subsp. *lactis*, BB-12 and *L. rhamnosus* GG on cultured Caco-2 cells mainly on barrier integrity and production of inflammatory mediators have been investigated [107].

In many cases, mechanisms and health effects of paraprobiotics are very similar to those exerted by probiotics in terms of stimulation of cytokine expression and activation of the cell signaling system, indicating that the loss of viability due to cell inactivation does not hamper beneficial functions. Reduction in the expression of proinflammatory cytokines (IL-8, TFN-α) and enhanced synthesis of anti-inflammatory cytokines (IL-10) is a key mechanism associated with beneficial effects on anti-inflammatory diseases. A switch in Th1/Th2 cell differentiation that involves the increased expression of IL-12 and IFN-γ and the decreased expression of IL4 and IL13 thus enhancing Th1 cell population is the basis of positive effects on allergies and atopic dermatitis treatment [16,22,108]. However, the possibility that live and inactivated cells lead to the same beneficial effect as a result of different mechanisms of action has also been observed [31].

In the last decade, evidence of beneficial effects of paraprobiotics in prophylaxis and treatment of several pathologies including diarrhea, colitis, respiratory, intestinal and liver diseases induced by alcohol, inflammation and allergies have been corroborated by in vivo studies in murine models and human clinical trials [10,27,108]. In this light, a particular effort was paid to investigate the effect of the administration of paraprobiotics in vulnerable people such as the elderly, as probiotics could present safety risks, especially for these subjects. The elderly could be more susceptible to infections and age-related inflammatory diseases and may exhibit dysfunctions in both innate (quantitative and functional changes in neutrophils, monocytes, dendritic and natural killer cells, including altered expression of TLRs and cytokines) and adaptive immune system (qualitative and quantitative alterations in T and B cell subpopulations) [109,110,111]. Therefore, efficacy of paraprobiotics assumes great importance for the elderly due to the advantages that they may offer in terms of both safety and production of novel functional foods that could be easily consumed by these subjects.

Short-lived senescence-accelerated mouse prone 1 (SAMP1) has been used as an animal model to investigate paraprobiotic effects on age-related immunosenescence. The ability of heat-killed *Lactobacillus gasseri* TMC0356 cells to increase the expression of IL-12 and interferon-α and -β receptor 1 and the activities of splenic natural killer cells demonstrated that oral administration of the paraprobiotic could modulate cell-mediated immunity [112]. In a similar animal model, a diet supplemented with heat-killed *Lactococcus lactis* G50 enhanced intestinal immunity and inhibited the growth of enteric H_2_S-producing bacteria [113]. The decrease of immunity represents a well-known consequence of ageing and may contribute to the vulnerability of the elderly to respiratory infections [110]. In a randomized placebo-controlled double-blind trial on elderly subjects, Kotani et al. demonstrated that the oral intake of heat-killed cells of *Lactobacillus pentosus* b240 induced an enhanced secretion of the salivary immunoglobulin A (sIgA) that plays a key role in mediating humoral mucosal immune response [91]. Moreover, oral intake of *L. pentosus* b240 exerted protective effects against the common cold in elderly adults (reducing its incidence rate) related to the ability of this paraprobiotic to improve mucosal immunity through enhanced secretion of sIgA. The elderly group following the supplemented diet also experienced an increased general well-being independently from common cold, thus suggesting other beneficial effects due to paraprobiotic consumption [92]. Interestingly, Murata et al. recently observed that a diet that includes the intake of heat-killed *Lactobacillus paracasei* MCC1849 cells caused similar effects (improved resistance to common cold infections and maintenance of a desirable mood, even under mental stress conditions) on a pre-specified subgroup of subjects of young healthy adults susceptible to common cold [81].

A strict relationship between the enhancement of immune functions and the improvement of health-related quality of life (QOL) prompted by heat-killed *Lactobacillus plantarum* L-137 cells has been previously shown in healthy adults including a limited subgroup of elderly subjects [93]. More recently, prolonged consumption of ADR-159 (a product containing co-fermented *Lactobacillus fermentum* and *Lactobacillus delbrueckii* subjected to an extensive high-temperature post-production treatment) has proved to positively affect social behavior and reduce baseline corticosterone levels (stress hormone) in healthy mice, suggesting potential anxiolytic and antidepressant effects of paraprobiotics [19].

As a matter of fact, anxiety, stress and depression are frequently encountered in the elderly and constitute additional risk factors for their well-being. In this frame, the possibility to use paraprobiotics as a natural aid to alleviate anxiety, stress and depression symptoms could be particularly important for the elderly, as they are more susceptible to drug-induced side effects.

In the last years, evidence of the beneficial impact that probiotics (prebiotics and synbiotics) could have on cognitive functions and neurological diseases via the microbiota–gut–brain axis are gaining increasing attention [114].

Although knowledge in this area remains limited and further studies are needed to definitively assess the health-promoting effects of paraprobiotics, these findings reinforce the idea of the potential impact of paraprobiotics for ameliorating general well-being, a perspective that could be particularly important for frail subjects.

## 7. Conclusions

Paraprobiotics represent an important opportunity for the development of innovative functional foods suitable for people with weak immunity. These products are also characterized by greater stability and can be stored without the cold chain, thereby facilitating industrial handling and wide commercialization.

However, there are several aspects that need to be defined and others on which the scientific community must do substantial work.

Probiotic cell inactivation may be achieved by physical or chemical procedures, which may alter cell structure and function, making bacteria lose their ability to grow and reproduce while preserving (or not) the beneficial effects exhibited by their viable counterparts. Thus, a further challenge is the use of appropriate methodologies to evaluate their biological activity and to identify the components responsible for the health effect. In addition, some challenges exist when considering food as a delivery vehicle for paraprobiotics, similarly to what happens for probiotics. Easy production, i.e., fast, controlled and affordable inactivation methods, good solubility into foods and limited interaction with food components are other aspects to be taken into account for practical application of paraprobiotics in foods. Their application must then necessarily go through the development of an approach able to correlate the biological response with the physical state and vitality of the microbial strain and to allow quality control of these products and regulatory interventions (“health claims”).

## Figures and Tables

**Table 1:** Health benefits of bioactive inactivated probiotic cells in food products and nutraceuticals as described in the article.

References	Microorganisms	Inactivation Treatment	Cell Concentration	Foods/ Nutraceuticals	Health Benefits
Nishida et al., 2017 [63]	*Lactobacillus gasseri CP2305*	Heat (95 °C for 30 s)	5.3 × 10^7^ CFU/g	Fermented milk beverage	Amelioration of stress-related symptoms and improvement of sleep quality
Sawada et al., 2016 [64]	*Lactobacillus gasseri* CP2305	Heat (69 °C for over 1 s + 95 °C for 30 s)	5.3 × 10^7^ CFU/g	Fermented milk beverage	Regulation of intestinal function
Nobutani et al., 2017 [65]	*Lactobacillus gasseri* CP2305		4.8 × 10^7^ CFU/g	Fermented milk	Alleviation of irritable bowel syndrome
Zeng et al., 2016 [66]	*Lactobacillus bulgaricus, Streptococcus thermophilus, Lactobacillus acidophilus*	Heat (water bath at 65 °C for 60 min)	1 × 10^7^–10^8^–10^9^ CFU/mL	Yoghurt	Prevention of epithelial barrier dysfunction
Berni Canani et al., 2017 [67]	*Lactobacillus paracasei* CBAL74	Heat (85 °C for 20 s)	5.9 × 10^11^ CFU/g	Spray-dried fermented milk	Modulation of gut microbiota
Liu et al., 2015 [68]	*Lactobacillus bulgaricus, Streptococcus thermophilus*	Heat (75 °C for 4 s)	1 × 10^7^–10^8^–10^9^ CFU/mL	Yoghurt	Improvement of constipation symptoms and intestinal health
Rodríguez–Figueroa et al., 2013 [69]	*Lactococcus lactis*	Heat (98 °C for 10 min)	1 × 10^4^–10^5^ CFU/mL	Fermented milk	Antihypertensive and hypolipidemic effects
Murata et al., 2018 [81]	*Lactobacillus paracasei* MCC1849	Heat–killed in water	1 × 10^10^–3 × 10^10^ CFU/mL	Cell powder	Improvement of resistance to common cold infections and maintenance of a desirable mood state
Buckley et al., 2018 [83] Mehling, Busjahn, 2013 [84]	*Lactobacillus reuteri* DSMZ17648	Spray-dried dead cells	5 × 10^9^ cells/tablet, four tablets daily	Solid tablets	Control of *Helicobacter pylori* in humans
Xiao et al., 2003 [86]	*Lactobacillus acidophilus* LB	Heat-killed, lyophilized	5 × 10^9^ cells/tablet, two tablets daily	Tablets	Clinical efficacy in the treatment of chronic diarrhea
Sawada et al., 2019 [87]	*Lactobacillus gasseri* CP2305	Heat-inactivated	1 × 10^10^ cells/200 mL	Sports drink	Faster recovery from fatigue and improvement of physical and mental stress-associated symptoms in athletes
Sugawara et al., 2016 [88]	*Lactobacillus gasseri* CP2305	Pasteurized at 90 °C and freeze-dried	1 × 10^10^ cells/200 mL	Beverage	Regulatory effect on gut environment and function
Nishida et al., 2017 [89]	*Lactobacillus gasseri* CP2305	Pasteurized at 90 °C and freeze-dried	1 × 10^10^ cells/200 mL	Beverage	Improvement of chronic stress-associated symptoms in healthy young adults
Barros et al., 2020 [90]	*Lactobacillus casei* 01	Ohmic heating (8 V/cm, 95 °C for seven min, 60 Hz)		Whey-grape juice drink	Control of postprandial glycemia in healthy adults
Kotani et al., 2010 [91]	*Lactobacillus pentosus* b240	Heat-killed (autoclave sterilization for 15 min)	6 × 10^9^ cells/125 mL	Water beverage	Acceleration of salivary immunoglobin A secretion in the elderly
Shinkai et al., 2013 [92]	*Lactobacillus pentosus* b240	Heat-killed (autoclave sterilization for 15 min)	2 × 10^9^ or 2 × 10^10^ cells/tablet	Tablets	Reduction of cold incidence trough mucosal immunity in the elderly
Hirose et al., 2006 [93]	*Lactobacillus plantarum* L-137	Heat-killed (70 °C for 10 min)	50 mg LP20 (20% paraprobiotic and 80% dextrin), one capsule daily	Gelatin capsules	Enhancement of acquired immunity and improvement of the quality of life in healthy subjects
